# Deep learning-based whole-body PSMA PET/CT attenuation correction utilizing Pix-2-Pix GAN

**DOI:** 10.18632/oncotarget.28583

**Published:** 2024-05-07

**Authors:** Kevin C. Ma, Esther Mena, Liza Lindenberg, Nathan S. Lay, Phillip Eclarinal, Deborah E. Citrin, Peter A. Pinto, Bradford J. Wood, William L. Dahut, James L. Gulley, Ravi A. Madan, Peter L. Choyke, Ismail Baris Turkbey, Stephanie A. Harmon

**Affiliations:** ^1^Artificial Intelligence Resource, National Cancer Institute, National Institutes of Health, Bethesda, MD 20892, USA; ^2^Molecular Imaging Branch, National Cancer Institute, National Institutes of Health, Bethesda, MD 20892, USA; ^3^Radiation Oncology Branch, National Cancer Institute, National Institutes of Health, Bethesda, MD 20892, USA; ^4^Urologic Oncology Branch, National Cancer Institute, National Institutes of Health, Bethesda, MD 20892, USA; ^5^Center for Interventional Oncology, National Cancer Institute, National Institutes of Health, Bethesda, MD 20892, USA; ^6^Genitourinary Malignancies Branch, National Cancer Institute, National Institutes of Health, Bethesda, MD 20892, USA; ^7^Center for Immuno-Oncology, National Cancer Institute, National Institutes of Health, Bethesda, MD 20892, USA

**Keywords:** deep learning, PSMA PET, attenuation correction

## Abstract

Purpose: Sequential PET/CT studies oncology patients can undergo during their treatment follow-up course is limited by radiation dosage. We propose an artificial intelligence (AI) tool to produce attenuation-corrected PET (AC-PET) images from non-attenuation-corrected PET (NAC-PET) images to reduce need for low-dose CT scans.

Methods: A deep learning algorithm based on 2D Pix-2-Pix generative adversarial network (GAN) architecture was developed from paired AC-PET and NAC-PET images. ^18^F-DCFPyL PSMA PET-CT studies from 302 prostate cancer patients, split into training, validation, and testing cohorts (*n* = 183, 60, 59, respectively). Models were trained with two normalization strategies: Standard Uptake Value (SUV)-based and SUV-Nyul-based. Scan-level performance was evaluated by normalized mean square error (NMSE), mean absolute error (MAE), structural similarity index (SSIM), and peak signal-to-noise ratio (PSNR). Lesion-level analysis was performed in regions-of-interest prospectively from nuclear medicine physicians. SUV metrics were evaluated using intraclass correlation coefficient (ICC), repeatability coefficient (RC), and linear mixed-effects modeling.

Results: Median NMSE, MAE, SSIM, and PSNR were 13.26%, 3.59%, 0.891, and 26.82, respectively, in the independent test cohort. ICC for SUV_max_ and SUV_mean_ were 0.88 and 0.89, which indicated a high correlation between original and AI-generated quantitative imaging markers. Lesion location, density (Hounsfield units), and lesion uptake were all shown to impact relative error in generated SUV metrics (all *p* < 0.05).

Conclusion: The Pix-2-Pix GAN model for generating AC-PET demonstrates SUV metrics that highly correlate with original images. AI-generated PET images show clinical potential for reducing the need for CT scans for attenuation correction while preserving quantitative markers and image quality.

## INTRODUCTION

PET-CT is a standard imaging modality in oncology for initial diagnosis [1–3]. Furthermore, follow-up PET-CTs can help to evaluate quantitative treatment response both clinically and in research/clinical trials [4]. Roughly half of the radiation exposure to patients is from the PET component whereas the remainder half from the CT component of the PET-CT, which is mainly utilized for attenuation correction of the PET signal [5]. The number and the frequency of PET-CT scans which patients can undergo during their oncologic care follow-up are limited by allowable annual radiation exposure. Reducing or eliminating the CT component of PET could reduce total radiation exposure by half [6, 7]. While some artificial intelligence (AI) methods for replacing the need for CT in PET-CT studies have been reported in the literature, primarily in FDG-PET studies [8–11], AI-based methodologies for post-acquisition attenuation correction of PET (AC-PET) have been researched in recent years mainly for PET-MR applications [12]. Generative-adversarial network (GAN) is a popular deep-learning approach for generating such images. In fact, prior studies have shown that GANs can successfully generate AC-PET from either a synthetic CT to create μ-maps [13, 14] or directly generate AC-PET from non-attenuation corrected PET (NAC-PET) [15], both with good similarity metrics compared to ground truths.

Prostate-specific membrane antigen (PSMA) targeted PET is a new molecular imaging method for identifying and quantifying sites of prostate cancer disease, especially for detecting biochemical recurrences after initial therapy [16, 17]. ^68^Ga-PSMA-11 and ^18^F-DCFPyL were recently approved as PET imaging agents by the US Food and Drug Administration [18, 19]. PSMA PET has demonstrated improved accuracy and specificity for recurrence detection over conventional imaging tools and alters clinical management in many patients [20]. Research on its effect on long-term patient outcomes is still ongoing [17].

In this study, we aim to use an image-to-image translation GAN AI tool to produce attenuation-corrected PET (AC-PET) images from non-attenuation-corrected PET (NAC-PET) images to reduce need for low-dose CT scans while maintaining image quality and quantitative standardized uptake value (SUV) metrics.

## RESULTS

**Figure 1:**
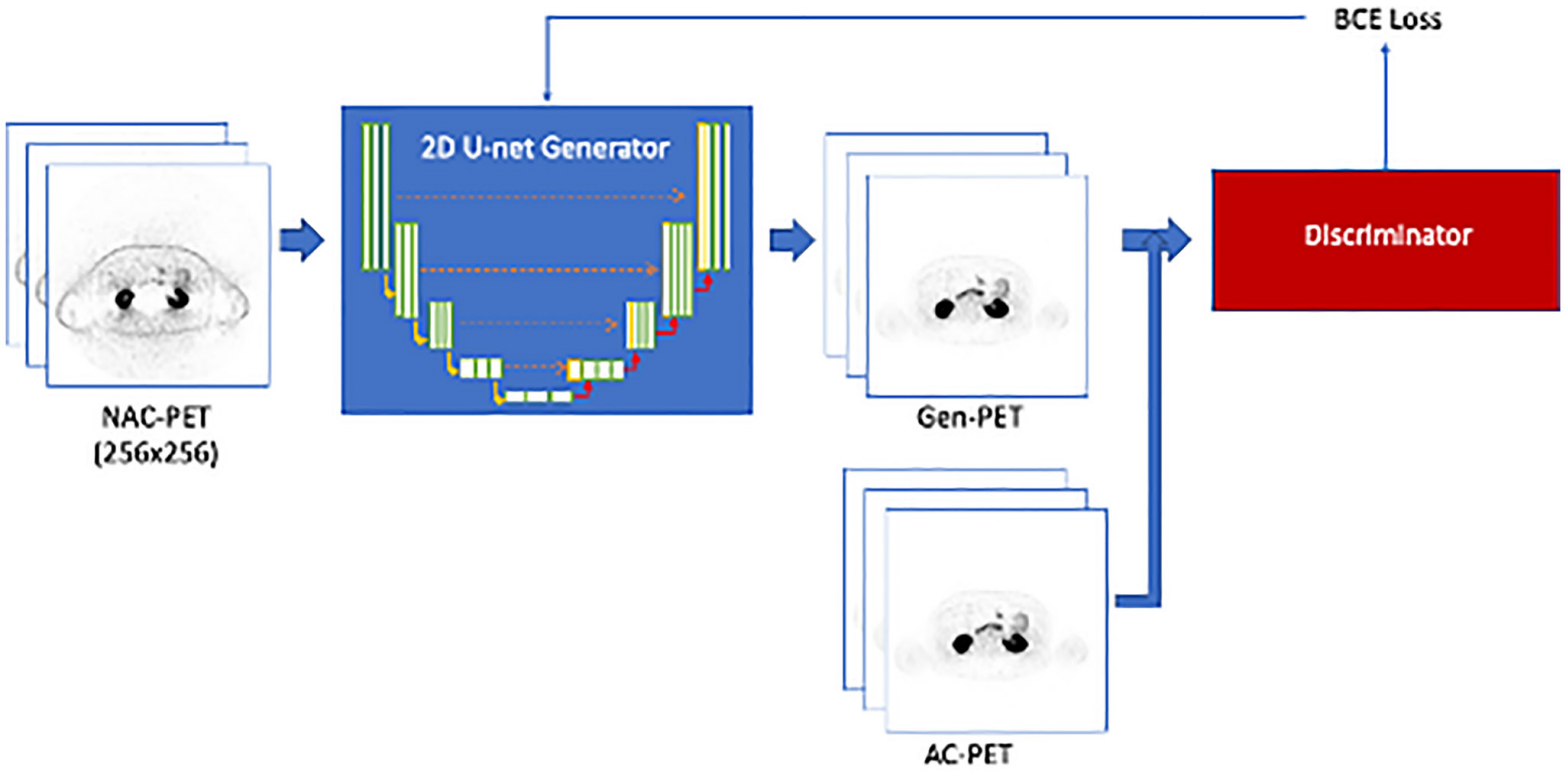
Training workflow for the Pix-2-Pix GAN model. The 2D U-net generator is trained to create Gen-PET from NAC-PET. The discriminator is used to classify between generated and real AC-PET, and the discriminator loss (BCE loss) is used to update generator training parameters.

Figure 1 shows the training input and workflow based on deep-learning model was based on 2D Pix-2-Pix GAN architecture [21]. The data cohort was divided into 183 AI model training studies, 60 validation studies during training, and 59 for independent testing studies. All splits were performed on the patient-level, i.e., patients with multiple scans were only included once in training, validation, or testing.

### Image-based AI model performance

Table 1 shows the image-based evaluation results of comparing the Gen-PET and the AC-PET from both models in validation and test datasets; model V1 represents the training using NAC-SUV to generate AC-SUV, and model V2 refers to the training with the Nyul-normalized NAC-SUV to generate the AC-SUV.

**Table 1:** Image-based evaluation results (median and range) for the Pix-2-Pix GAN models

Data cohort	AI model	NMSE (%)	PSNR (dB)	MAE (%)	SSIM
Validation	V1	9.54 (2.09, 48.39)	27.026 (19.568, 29.286)	2.50 (1.84, 4.71)	0.954 (0.905, 0.970)
	V2	13.40 (4.89, 80.87)	26.419 (18.241, 58.423)	3.73 (3.13, 6.87)	0.733 (0.684, 0.752)
Test	V1	13.26 (3.44, 257.37)	26.819 (16.777, 29.211)	3.59 (2.22, 7.25)	0.891 (0.840, 0.911)
	V2	13.334 (3.46, 292.46)	27.122 (16.999, 29.453)	3.36 (2.20, 7.02)	0.951 (0.910, 0.969)

**Figure 2:**
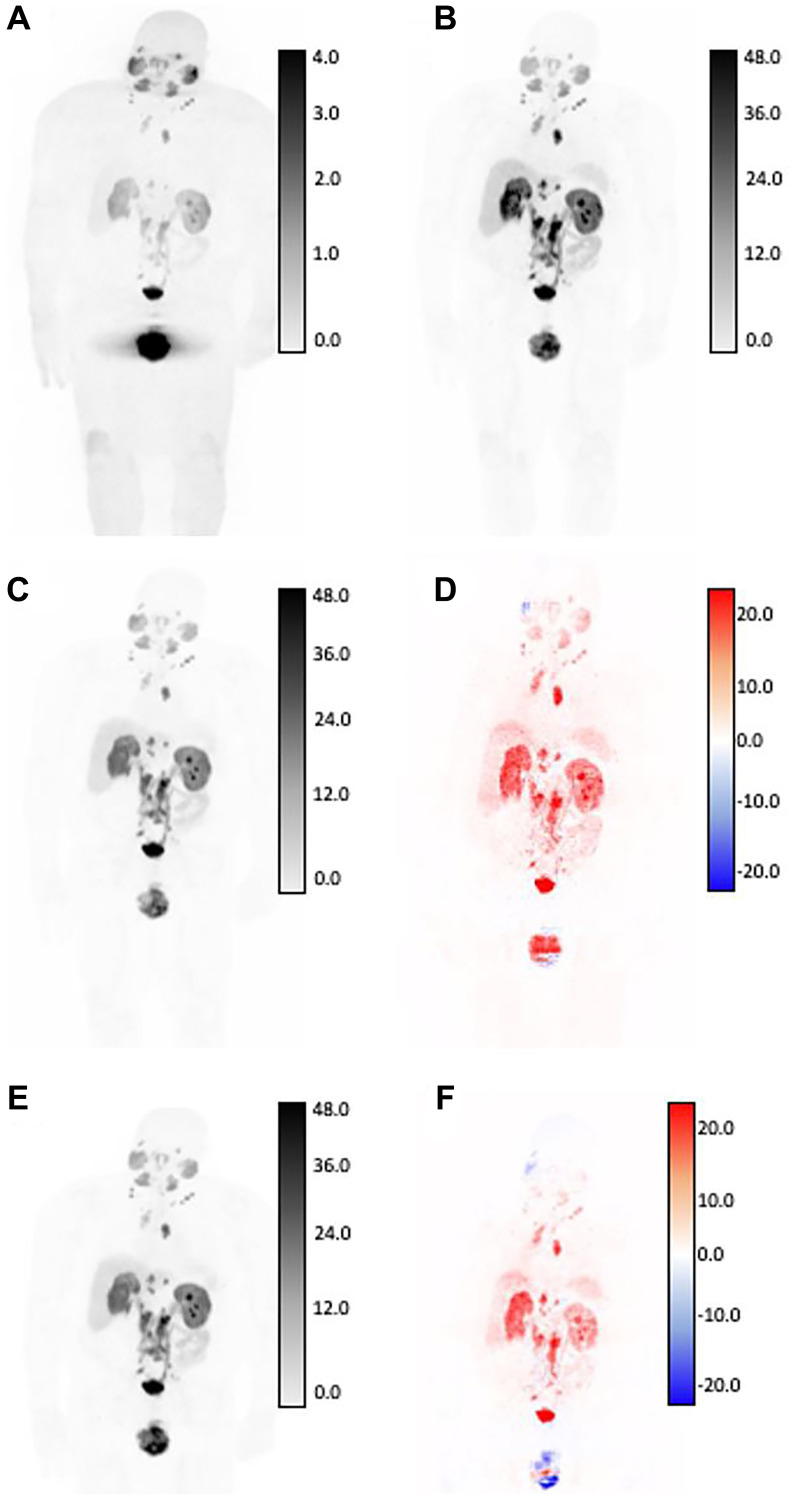
SUV differences between GAN outputs and original AC-PET, shown in coronal view for patient with high tumor burden by expert assessment. Original NAC-PET used as input to GAN algorithms (**A**) and associated original AC-PET (**B**). AI-generated PET from V1 normalization strategy (**C**) and calculated difference map (**D**). AI-generated PET from V2 normalization strategy (**E**) and calculated difference map (**F**). Bottom row: V2-generated PET (left) and difference map between v2 and AC-PET (right). For AC-original, V1, and V2 SUV maps, all images are shown on identical SUV scale. Difference maps reflect absolute error from original SUV (red = underestimation of SUV by AI, blue = overestimation of SUV by AI). Larger SUV differences were observed in areas of high normal-tissue tracer uptake, including: kidneys, ureters, bladder, and salivary glands.

**Figure 3:**
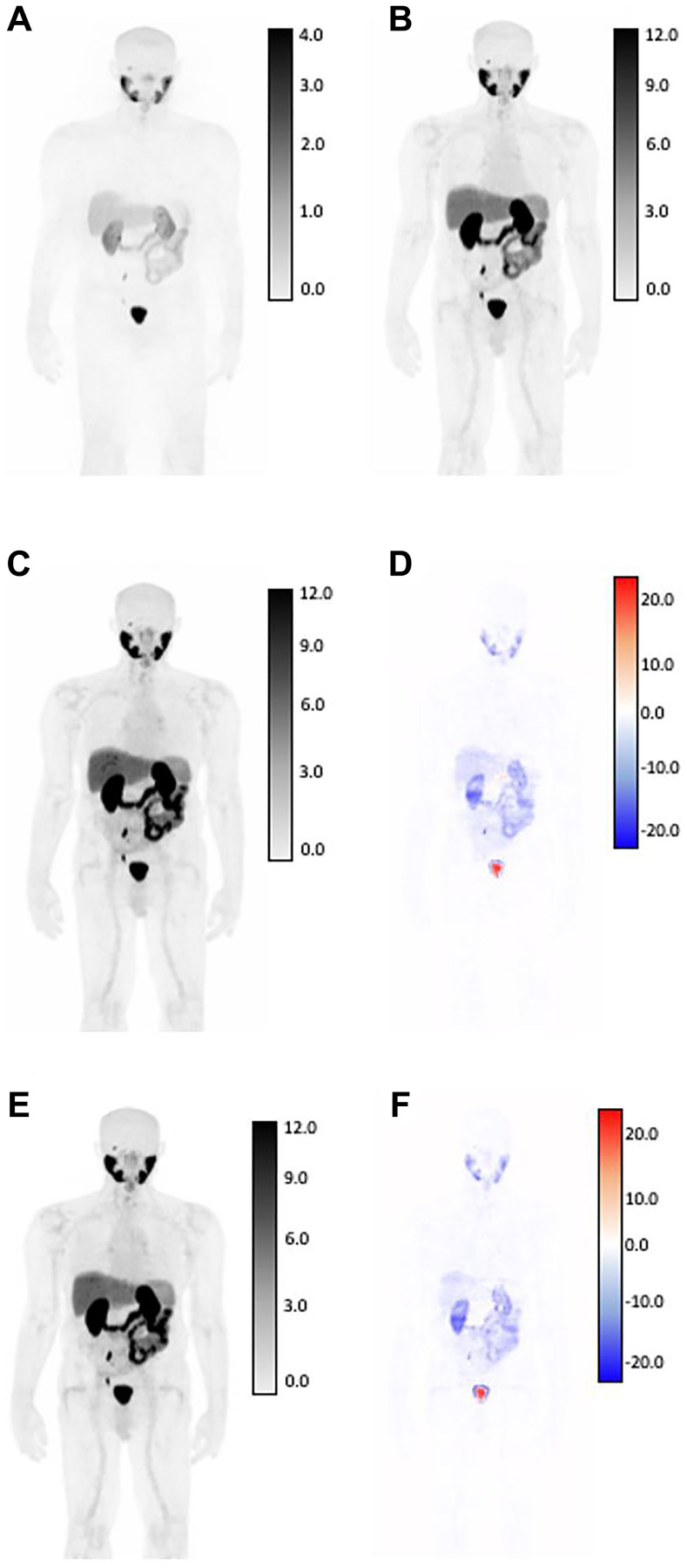
SUV differences between GAN outputs and original AC-PET, shown in coronal view for patient with low tumor burden by expert assessment. Original NAC-PET used as input to GAN algorithms (**A**) and associated. Original AC-PET (**B**). AI-generated PET from V1 normalization strategy (**C**) and calculated difference map (**D**). AI-generated PET from V2 normalization strategy (**E**) and calculated difference map (**F**). Bottom row: V2-generated PET (left) and difference map between v2 and AC-PET (right) For AC-original, V1, and V2 SUV maps, all images are shown on identical SUV scale. Difference maps reflect absolute error from original SUV (red = underestimation of SUV by AI, blue = overestimation of SUV by AI). Larger SUV differences were observed in areas of high normal-tissue tracer uptake, including: kidneys, ureters, bladder, and salivary glands.

Median scan-level NMSE of the validation set was used to select the best model checkpoint during the training process, which was found to be 9.54% and 13.4%, respectively. Among the independent testing cohort, the median NMSEs for V1 and V2 were 13.26% and 13.33%. Median MAEs for V1 and V2 GAN models within the test cohort were 3.59% and 3.36%, median SSIMs were 0.891 and 0.951, and median PSNRs for both GAN models were 26.82 and 27.12. Figures 2 and 3 show two generated images from the testing dataset, qualitatively demonstrating areas of highest discordance (i.e., relative difference from AC-PET) in high-uptake regions corresponding to normal anatomical uptake distribution, such as kidneys and bladder. Figure 2 shows a patient with a large number of visible lesions, and Figure 3 shows a patient with a small number of visible lesions.

### Lesion-based AI model performance

**Figure 4:**
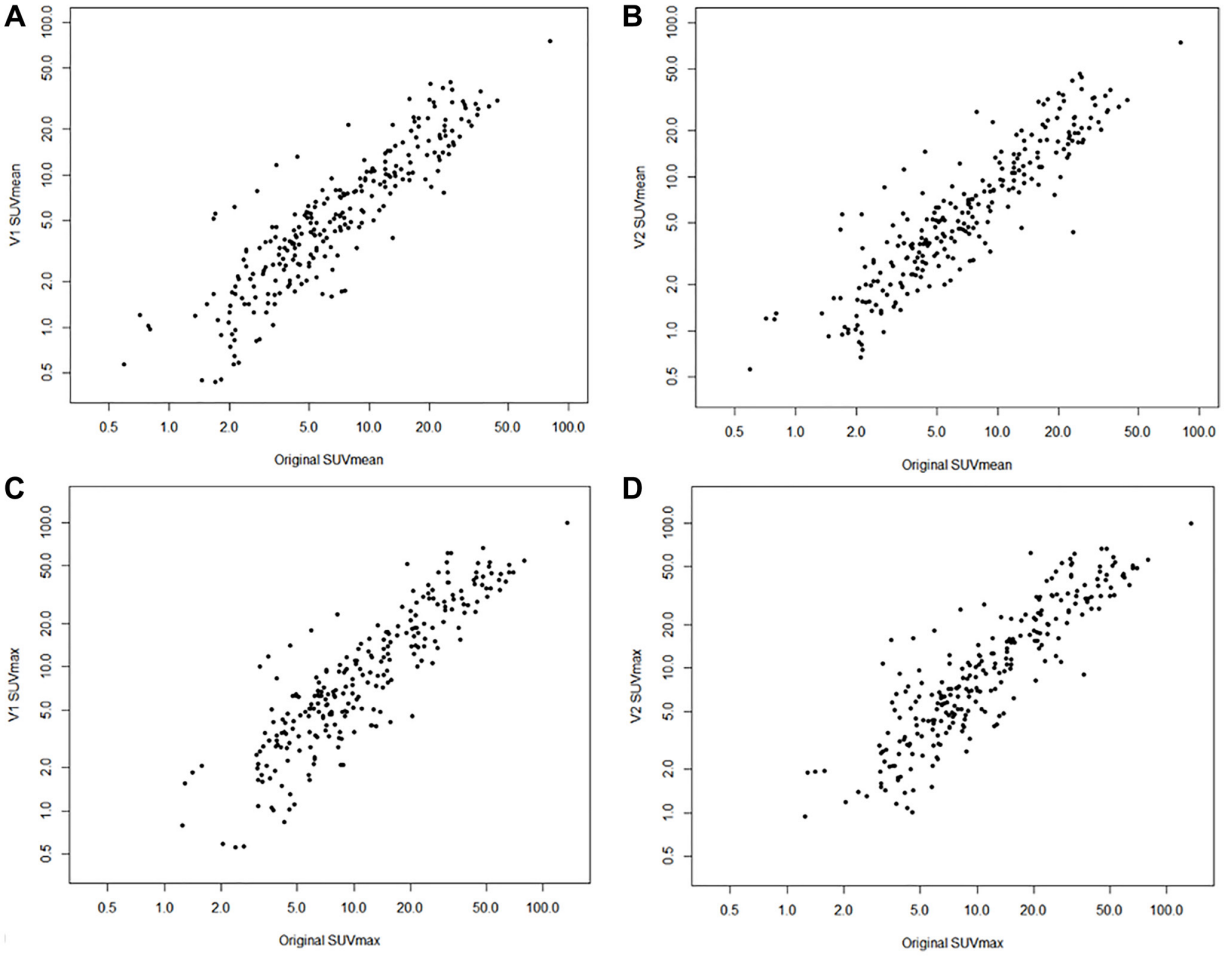
Scatter plots of original SUVs and AI-generated SUVs. SUV_mean_ of original and V1 model (**A**) and V2 model (**B**). SUV_max_ of original and V1 model (**C**) and V2 model (**D**). Note all axes plotted on log scale.

**Figure 5:**
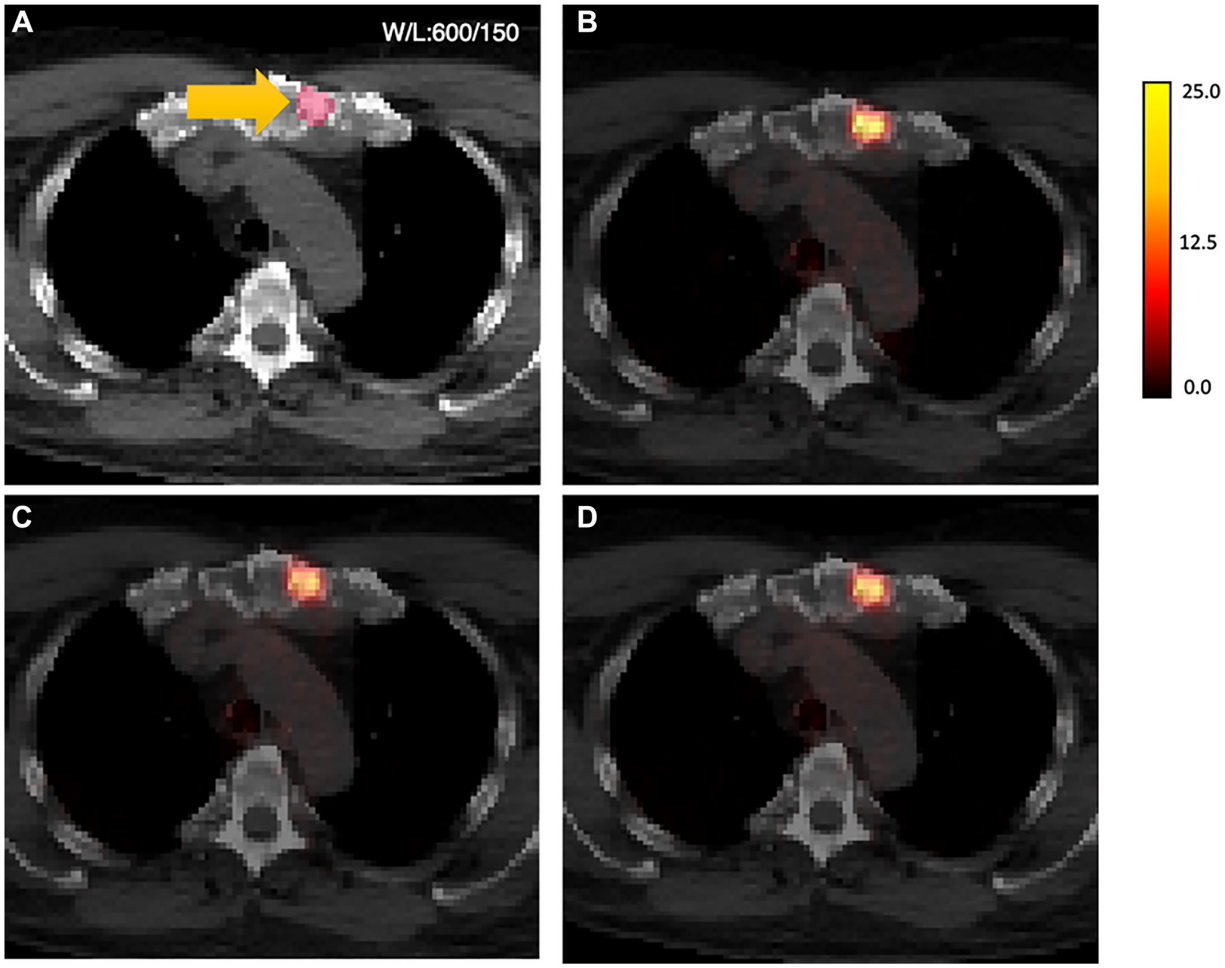
AI-generated PET results shown overlaid on CT. (**A**) original CT images, sternum)lesion contour in yellow, (**B**) original AC-PET overlaid on CT (SUV_max_ = 23.45, SUV_mean_ = 12.18); (**C**) V1-PET on CT (SUV_max_ = 19.80, SUV_mean_ = 10.11); (**D**) V2-PET on CT (SUV_max_ = 20.06, SUV_mean_ = 10.57). Note: CT images were resampled to the voxel resolution of the PET images and shown in unenhanced formatting (no smoothing) for voxel-based visual comparison between methods.

Of the 59 studies in the test cohort, 43 patients had a total of 259 positive lesions categorized as PSMARADS >4. Table 2 shows the data breakdown by lesions, with the majority occurring in lymph nodes (58%), followed by bones (29%) and intra-prostate (12%). No significant differences were observed for SUV metrics for either model compared to the original SUV metrics defined by AC-PET by paired Wilcoxon testing (Table 3). Table 4 reports repeatability metrics calculated from relative difference (%) from AC-PET for both methods. ICC was observed to be similar for both V1 (SUV_mean_ ICC = 0.895, SUV_max_ ICC = 0.880) and V2 (SUV_mean_ ICC = 0.888, SUV_max_ ICC = 0.880), respectively, reflecting high agreement with AC-PET SUV metrics. While the bias in both SUV_mean_ and SUV_max_ was higher for the V1 method compared to the V2 method, the overall RC was lower for the V1 method, reflecting modestly better agreement (Table 4). Figure 4 shows the scatter plot of SUV_max_ and SUV_mean_ comparisons between the original AC-PET images and the AI-generated PET images. A representative image of lesion-based uptake differences, selected as lesion nearest to mean bias from Table 4, is shown in Figure 5. Additional representative images from 1st quartile of mean bias and 4th quartile of mean bias are shown in Supplementary Figures 1 and 2, respectively.

**Table 2:** Data breakdown of lesions included in evaluation of differences between original and generated PET

Variable		Median (range) or *n*
*n* lesions per patient		2 (1–56)
PSMA RADS	4	65
	5	194
Location	Lymph node	151
	Bone	74
	Prostate	32
	Other	2
HU_mean_		9.3 (−825, 565)

**Table 3:** Comparison of SUV metrics derived from physician contours

	Original	V1	V2	*p*-value, V1	*p*-value, V2
SUV_max_	9.985 (1.246, 134.246)	8.14 (0.564, 99.9)	8.93 (0.941, 99.9)	0.3297	0.7598
SUV_mean_	6.454 (0.593, 80.470)	5.23 (0.437, 75.3)	5.28 (0.654, 74.3)	0.1758	0.9591

*p*-values are from Wilcoxon signed rank test for clustered data to test differences in SUVs between AC-PET and Gen-PET.

**Table 4:** Repeatability metrics for AI-generated PET

Method	Metric	B (%)	stdev (%)	RC (%)	ICC
v1	SUV_mean_	13.15	22.02	61.04	0.895
	SUV_max_	14.85	23.20	64.31	0.880
v2	SUV_mean_	5.89	24.02	66.58	0.888
	SUV_max_	6.33	26.84	74.40	0.880

Bias reported as mean relative difference from original AC-PET. Standard deviation (stdev), Repeatability Coefficient (RC), and Intraclass Correlation Coefficient (ICC) determined from mixed effects model accounting for clustered data.

Several lesion-based features were shown to have a significant relationship when evaluating the relative difference of Gen-PET SUV metrics to AC-PET SUV metrics (Table 5). For both models, Gen-PET models had lower overall error in lesions with higher uptake, as determined both by the original SUVs and by PSMA-RADS categorization. CT-based density measured by HU_max_ was also trending (for V2 SUV_max_) or significant (for V1 SUV_mean_, V1 SUV_max_, V2 SUV_mean_), reflecting that higher error was observed in more dense lesions (Table 5). This is further corroborated when evaluating anatomical location, which demonstrated that the non-LN lesions (i.e., bone or intraprostatic lesions) had higher error with Gen-PET quantitation.

**Table 5:** Linear mixed-effects models for assessing impact of lesion features on relative difference in SUV metrics

Model	SUV metric	Fixed effect		beta	std error	*t* statistic	*p*-value
V1	SUV_mean_	Base					ref
		Original SUV_mean_		−0.636	0.172	−3.71	2.56E-04
		HU_mean_		0.020	0.014	1.42	0.154
		HU_max_		1.20E-04	5.69E-05	2.11	0.035
		Volume		−0.128	0.122	−1.04	0.296
		Location	Bone	6.79	4.99	1.36	0.075
			Other	30.9	17.7	1.75	
			Prostate	12.2	6.35	1.92	
		PSMARADS	5	−8.20	3.80	−2.16	0.032
	SUV_max_	Base					ref
		Original		−0.384	0.101	−3.80	1.78E-04
		HU_mean_		0.027	0.014	1.85	0.065
		HU_max_		0.014	0.006	2.36	0.019
		Volume		−0.141	0.129	−1.10	0.273
		Location	Bone	11.4	5.22	2.19	0.060
			Other	32.1	18.5	1.73	
			Prostate	6.38	6.63	0.962	
		PSMARADS	5	−10.9	3.97	−2.74	0.007
V2	SUV_mean_	Base					ref
		Original SUVmean		−0.410	0.190	−2.15	3.20E-02
		HU_mean_		0.023	0.015	1.55	0.120
		HU_max_		0.013	0.006	2.06	0.040
		Volume		−0.134	0.133	−1.01	0.314
		Location	Bone	9.21	5.40	1.71	0.084
			Other	37.9	19.2	1.97	
			Prostate	7.47	6.86	1.09	
		PSMARADS	5	−9.56	4.13	−2.32	0.021
	SUV_max_	Base					ref
		Original		−0.252	0.119	−2.12	3.51E-02
		HU_mean_		0.019	0.017	1.14	0.253
		HU_max_		0.011	0.007	1.53	0.127
		Volume		−0.147	0.149	−0.986	0.324
		Location	Bone	10.4	6.00	1.73	0.059
			Other	39.7	21.4	1.86	
			Prostate	−0.313	7.61	−0.411	
		PSMARADS	5	−7.92	4.63	−1.71	0.088

Base model: Rel.Diff ~ (1|patient). Evaluated models: Rel.Diff ~ <variable> + (1|patient). Statistical significance relative to base model determined from ANOVA.

## DISCUSSION

A Pix-2-Pix image translation GAN was developed to generate AC- PET from NAC-PET for ^18^F-DCFPyL PSMA whole-body scans. Gen-PET was evaluated quantitatively with image comparison metrics, including pixel-wise error rates and structural similarities. Additional quantitative evaluation included lesion-based standard uptake value repeatability metrics, which are measured by the SUV_max_ and SUV_mean_ of lesions contoured by expert physicians. Overall, the image-based errors were relatively low and approaching clinically acceptable limits of agreement according to previously reported FDG PET studies.

Two models were created and evaluated: one with the standard preprocessing step of converting the NAC-PET to SUV before training (V1), and the other one adding a Nyul normalization step during standard preprocessing (V2). According to image comparison metrics, the performance of the two models was very similar to each other, with V2 model having a slightly better performance in SSIM and MAE, and V1 performing better in NMSE and PSNR. While no similar work has been previously reported on AI-generated PSMA PET domain, the SSIM and PSNR metrics of our GAN model show comparable results to previous studies with FDG PET and synthetic CT. Tao et al. [22] reported an SSIM of 0.9 and a PSNR of 29.35 while comparing GAN-created synthetic CT with brain MR. Dong et al. [15] reported a PSNR of 44.3. In addition, Dong et al. reported an NMSE of 1.21%, and Tao et al. reported an error rate of 17.46%. Potential reasons for higher NMSE metrics in our study could be due to differences in tracer and image acquisition, specifically due to the high dynamic range of SUV using ^18^F-DCFPyL. Areas of highest voxel-wise difference from the Gen-PET models and the original AC-PET could be observed specifically in the kidneys and bladder, where SUV uptake commonly ranges >100 and would not be captured by algorithm due to pre-processing thresholds to 100 SUV.

From lesion-based repeatability studies, V2 results showed slightly less mean difference in SUVs, but V2 results had slightly higher RC. We have not identified any study which used repeatability metrics in assessing GAN-generated PET; therefore, we compared our results with prior PSMA test-retest repeatability studies. In a test-retest repeatability study for ^18^F-DCFPyL by Jansen et al. [3], bias for SUV_max_ and SUV_mean_ was 1.9% and 1.0%, respectively. RCs for SUV_max_ and SUV_mean_ were 31% and 24.4%, respectively. Seifert et al. [2] reported RCs of lesion SUV_max_ and SUV_mean_ of 34.% and 32.7%, respectively, using ^68^Ga-PSMA-HBED-CC PET-CT scan. Future work is needed to determine whether the described Gen-PET methods would be within reliability metrics for response assessment criteria, such as PERCIST [23]. However, limited longitudinal data were available in the present study, and the clinical utility of PSMA PET/CT imaging in treatment response assessment is uncertain due to complex biological relationships between PSMA and standard-of-care androgen-deprivation therapies [24].

Examining our statistical results independently, we found that our *p*-values (>0.05) from the Wilcoxon test showing the AI-generated SUV metric are not statistically different from the original SUVs. We have found that SUV_mean_ has higher repeatability metrics than SUV_max_, which agrees with prior research in repeatability studies [3, 25, 26]. Results from repeatability evaluation confirm that the quantitative diagnostic measure of SUV does not change significantly from the AI-generated AC-PET. We also performed ANOVA to assess stand-alone factors that impact the AI-generated images. These factors include AC-PET SUV_mean_, AC-PET SUV_max_, HU_mean_, HU_max_, lesion location (prostate, lymph node, bone, and organ), lesion volume, and PSMA RADS (4 and 5). We found that higher uptake values correlate with lower bias, which can be corroborated with Werner et al. [26]. Higher HU_max_ (i.e., areas of higher attenuation/scatter errors) significantly correlates with bias, which is also represented in higher bias for lesions in bone versus lymph nodes. These results suggest that future research is needed on how to improve Gen-PET methods accounting for anatomical density (HU) and proximity to areas of clearance uptake (prostate) is warranted.

The results of our study indicate that AC-PET data can be generated from NAC-PET data using our Pix-2-Pix GAN model for ^18^F-DCFPyL PSMA-targeting PET imaging without using an attenuation correction CT. Such an AI-assisted strategy may help to reduce the radiation dose exposure related to attenuation correction CT acquisition in prostate cancer patients. While our method has such an advantage, the disadvantage is having no access to low-dose CT images during PSMA PET evaluations. This can be compensated for by use of staging diagnostic CT scans which are frequently required to be obtained prior to PSMA PET imaging by established cancer care guidelines. The staging CT scans can be registered to AI-assisted AC-PET data for diagnostic evaluations on PACS workstations.

Limitations of this study include the fact that we only assessed one type of PSMA tracer (^18^F-DCFPyL) from a single scanner in the dataset. While the homogeneity of the data protocol is important for initial training of the AI model, variety in training data (such as tracers, modalities, patient population, etc.) could make the model more robust. A larger collection of data in training and testing can also increase model performance. An evaluation of intra-subject SUV changes from two time points between the original AC-PET and the Gen-PET was considered, but ultimately was not viable due to insufficient data. Additionally, since we did not conduct a reader study in AI-model based AC-PET data, potential false-positive predictions created during image generation was not evaluated. However, we aim to evaluate the interaction between the nuclear medicine physicians and the AI-model based AC-PET in larger, independent validation cohorts. Finally, the GAN was trained to generate the AC-PET only, one of our future goals is to generate the synthetic CT for anatomical mapping purposes. Within our study population, we did not have a high representation of cases with CT-based artifacts, such as prosthetics, ports, or medical hardware which may influence the accuracy of attenuation correction. Future work and validation is needed to evaluate the performance of the model in these challenging clinical scenarios.

We have developed a Pix-2-Pix GAN model to perform attenuation correction on whole-body PSMA PET images with ^18^F-DCFPyL. The model successfully generates AC-PET images with high PSNR and low MAE. A repeatability study from segmented lesions shows reasonable reproducibility of SUV_max_ and SUV_mean_ between the original AC-PET and the generated AC-PET. AI-generated PET images has clinical potential for reducing the need for CT scans for attenuation correction while preserving quantitative markers and image quality in prostate cancer patients.

## MATERIALS AND METHODS

### Study population, image acquisition and annotation

^18^F-DCFPyL PET/CT imaging was performed in 283 consecutive patients (*n* = 302 scans) with histologically confirmed prostate cancer, including high-risk localized disease, biochemically recurrent disease, or suspected and/or known metastatic disease. All patients gave informed consent prior to participation in one and/or both clinical trials (BLINDED FOR PEER REVIEW). ^18^F-DCFPyL was synthesized under good manufacturing practices as previously described [27]. Patients were injected with 271.10 ± 38.00 MBq of ^18^F-DCFPyL and whole-body images were acquired after a 2-hour uptake period (3 min/bed position) using a 3D time of flight (TOF) GE Discovery MI DR scanner, with a 20-cm coronal and a 70-cm axial field of view. Image reconstruction used an AC 3D iterative MLEM algorithm using 29 subsets, 3 iterations, TOF, point spread function regularization parameter 6.0, and a Gaussian post-filter with 4.1-cm kernel, producing a final voxel resolution of 2.73 × 2.73 × 3.27 mm^3^. A low-dose non-contrast CT (120 kV, 60 mAs) was acquired with each PET scan for attenuation correction and anatomical co-registration purposes. For all studies, the non- attenuation-corrected PET (NAC-PET) and attenuation-corrected PET (AC-PET) were collected for analysis.

In the testing set, previously delineated volumetric contours of suspicious lesions were extracted for statistical analysis. Briefly, contours were drawn by two experienced nuclear medicine physicians (five years of experience reading PSMA PET/CTs) using a MIM workstation (version 6.9.2; MIM Software Inc.) based on study analysis completed in previously published research [28–30]. Only lesions highly suggestive of prostate cancer by consensus were included, i.e., those lesions matching PSMA Reporting and Data System (PSMA-RADS) category 4 or 5 [31]. All DICOM-RT structures for lesions in the testing cohort meeting inclusion criteria were extracted and converted to binary masks of lesion volumes for performance analysis.

### Image processing for AI model development

All PET and CT images were converted from the original DICOM format to NIFTI, with CT images resampled to PET image resolution for visualization purposes. Acquired in-plane resolutions were maintained (i.e., no resampling), reflecting 256 × 256 voxels in x-y directions, and number of slices in the z direction is kept consistent for all three series acquired within the same study session (range 299–623 slices in PET studies). PET studies were converted to SUVs based on DICOM header information to avoid compounding factors such as patient weight, radiotracer concentration, and scan time [32]. AC-PET images were clipped to an upper threshold of 100 before scaling to range (0, 1) for algorithm training.

Two methods were evaluated for NAC-PET pre-processing. First, NAC-PET images were clipped and scaled using the identical procedure as AC-PET described above (SUV threshold = 100). Second, a Nyul-based normalization step [33] was implemented on NAC-PET images to evaluate improvement in training due to uncertainties and variabilities in attenuation maps. Briefly, a set of 20 landmarks was obtained from each image of the training cohort, taken at equal increments from 80th to 99th percentiles. The mean landmarks obtained from all NAC-PET images in the training set were used to define the standard histogram for piece-wise normalization of all NAC-PET images. The mean landmarks from the training set were applied to both validation and testing datasets. Resultant images from both with and without Nyul-normalization models were compared.

### Deep-learning-based AI model development

The deep-learning model was based on 2D Pix-2-Pix GAN architecture for direct image-to-image translation [21]. The model consisted of two separate networks: the generator and discriminator. Figure 1 shows the training input and workflow.

Briefly, the generator architecture was based on a 2D U-net convolutional neural network [34], a well-documented and widely used network in biomedical image deep-learning solutions. The defining U-net feature, a skip connection between corresponding encoding and decoding layers in the model, was emphasized to preserve lower-level spatial information in direct image-to-image translation [35]. The generator was trained with paired PET slices, with NAC-PET as input and AC-PET as target at size 256 × 256. The discriminator network used the PatchGAN architecture, a convolutional neural network consisting of three layers [21]. The discriminator operated within 30 × 30 pixel patches from the input image with kernel size = 4, stride size = 2, and padding width = 1. Responses from all patches in the image were averaged to produce the ultimate discriminator output per input image (i.e., 2D slice). The discriminator loss is calculated from binary cross-entropy loss. The generator loss was calculated from a combination of discriminator loss and Least Absolute Deviations (L1) loss from generated and original images [21].

During the model training, studies in the training cohort were loaded into the model in a random order. The slices were fed into the training step in batches of 25 slices. Loss values from each batch within a single study were aggregated, and the overall training loss of each epoch was calculated by averaging aggregated loss from all studies. The validation step used mean square error as the metric for model evaluation. The model with the lowest averaged normalized mean squared error (NMSE) among validation data was saved as the final model.

The GAN model was prototyped in Python 3.8, including PyTorch 1.12 and MONAI 0.9 [36]. The model was trained with learning rate of 0.0002, Adam optimizer, in 25 epochs.

### Statistical analysis

At inference, generated AC-PET output images were multiplied by 100 to translate to SUV. Image-based and lesion-based methodologies were used to evaluate GAN-generated AC-PET (Gen-PET): image-based and lesion-based.

For image-based evaluation, the whole image was evaluated for pixel-wise value differences and structural similarities between AC-PET and Gen-PET. Image-based evaluation metrics included NMSE, mean absolute error (MAE), peak signal-to-noise ratio (PSNR), and structure similarity index (SSIM). Data were reported separately for validation and testing datasets.

Lesion-based analysis was performed within physician-derived volumes to assess the quantitative performance of the generated SUV metrics in Gen-PET compared to the original SUV metrics derived from the acquired AC-PET. For each lesion contour, maximum of SUV (SUV_max_) and mean SUV (SUV_mean_) were calculated on both the original AC-PET and the Gen-PET. Paired Wilcoxon signed-rank test was used to assess differences between original and generated SUV metrics using the Rosner–Glynne–Lee method to account for intrapatient correlation [37]. The lesion-based intraclass correlation coefficient (ICC) and repeatability coefficient (RC) were estimated from a mixed effect model of the relative difference in measurements with nested random effects for each SUV metric. Bias was reported as the mean relative difference between Gen-PET- and AC-PET-derived SUV metrics, calculated for each metric as relative difference = (AC-PET – Gen-PET)/AC-PET. Various fixed effects were subsequently added to the mixed effects model, such as AC-PET uptake value, Hounsfield unit (HU) summary statistics, lesion volume, lesion anatomic location, and PSMA-RADS score to assess their impact on relative difference in SUVs. Statistical testing of these models in comparison to a baseline model were completed using ANOVA. All *p*-values corresponded to two-sided tests, and any *p*-value < 0.05 was considered to represent a significant difference between results. Statistical analysis was completed using R (version 3.6.2).

## SUPPLEMENTARY MATERIALS



## Abbreviations

PET: Positron Emission Tomography
CT: Computed Tomography
AC: Attenuation Corrected
NAC: Non-Attenuation Corrected
GAN: Generative Adversarial Network
SUV: Standardized Uptake Value
NMSE: normalized mean square error
MAE: mean absolute error
SSIM: structural similarity index
PSNR: peak signal-to-noise ratio
ICC: intraclass correlation coefficient
RC: repeatability coefficient
AI: artificial intelligence
PSMA: Prostate-specific membrane antigen
PSMA-RADS: PSMA Reporting and Data System
HU: Hounsfield unit

## References

[1] Bai B , Bading J , Conti PS . Tumor quantification in clinical positron emission tomography. Theranostics. 2013; 3:787–801. 10.7150/thno.5629. 24312151 PMC3840412

[2] Seifert R , Sandach P , Kersting D , Fendler WP , Hadaschik B , Herrmann K , Sunderland JJ , Pollard JH . Repeatability of ^68^Ga-PSMA-HBED-CC PET/CT-Derived Total Molecular Tumor Volume. J Nucl Med. 2022; 63:746–53. 10.2967/jnumed.121.262528. 34446454 PMC9051594

[3] Jansen BHE , Cysouw MCF , Vis AN , van Moorselaar RJA , Voortman J , Bodar YJL , Schober PR , Hendrikse NH , Hoekstra OS , Boellaard R , Oprea-Lager DE . Repeatability of Quantitative ^18^F-DCFPyL PET/CT Measurements in Metastatic Prostate Cancer. J Nucl Med. 2020; 61:1320–25. 10.2967/jnumed.119.236075. 31924729 PMC7456167

[4] Violet J , Sandhu S , Iravani A , Ferdinandus J , Thang SP , Kong G , Kumar AR , Akhurst T , Pattison DA , Beaulieu A , Mooi J , Tran B , Guo C , et al. Long-Term Follow-up and Outcomes of Retreatment in an Expanded 50-Patient Single-Center Phase II Prospective Trial of ^177^Lu-PSMA-617 Theranostics in Metastatic Castration-Resistant Prostate Cancer. J Nucl Med. 2020; 61:857–65. 10.2967/jnumed.119.236414. 31732676 PMC7262220

[5] Devine CE , Mawlawi O . Radiation safety with positron emission tomography and computed tomography. Semin Ultrasound CT MR. 2010; 31:39–45. 10.1053/j.sult.2009.09.005. 20102694

[6] Kaushik A , Jaimini A , Tripathi M , D’Souza M , Sharma R , Mishra AK , Mondal A , Dwarakanath BS . Estimation of patient dose in (18)F-FDG and (18)F-FDOPA PET/CT examinations. J Cancer Res Ther. 2013; 9:477–83. 10.4103/0973-1482.119354. 24125986

[7] Khamwan K , Krisanachinda A , Pasawang P . The determination of patient dose from (18)F-FDG PET/CT examination. Radiat Prot Dosimetry. 2010; 141:50–55. 10.1093/rpd/ncq140. 20400773

[8] Burger C , Goerres G , Schoenes S , Buck A , Lonn AH , Von Schulthess GK . PET attenuation coefficients from CT images: experimental evaluation of the transformation of CT into PET 511-keV attenuation coefficients. Eur J Nucl Med Mol Imaging. 2002; 29:922–27. 10.1007/s00259-002-0796-3. 12111133

[9] Visvikis D , Lambin P , Beuschau Mauridsen K , Hustinx R , Lassmann M , Rischpler C , Shi K , Pruim J . Application of artificial intelligence in nuclear medicine and molecular imaging: a review of current status and future perspectives for clinical translation. Eur J Nucl Med Mol Imaging. 2022; 49:4452–63. 10.1007/s00259-022-05891-w. 35809090 PMC9606092

[10] Arabi H , AkhavanAllaf A , Sanaat A , Shiri I , Zaidi H . The promise of artificial intelligence and deep learning in PET and SPECT imaging. Phys Med. 2021; 83:122–37. 10.1016/j.ejmp.2021.03.008. 33765602

[11] Wang T , Lei Y , Fu Y , Curran WJ , Liu T , Nye JA , Yang X . Machine learning in quantitative PET: A review of attenuation correction and low-count image reconstruction methods. Phys Med. 2020; 76:294–306. 10.1016/j.ejmp.2020.07.028. 32738777 PMC7484241

[12] Chen Y , An H . Attenuation Correction of PET/MR Imaging. Magn Reson Imaging Clin N Am. 2017; 25:245–55. 10.1016/j.mric.2016.12.001. 28390526 PMC5385843

[13] Torrado-Carvajal A , Vera-Olmos J , Izquierdo-Garcia D , Catalano OA , Morales MA , Margolin J , Soricelli A , Salvatore M , Malpica N , Catana C . Dixon-VIBE Deep Learning (DIVIDE) Pseudo-CT Synthesis for Pelvis PET/MR Attenuation Correction. J Nucl Med. 2019; 60:429–35. 10.2967/jnumed.118.209288. 30166357 PMC6910626

[14] Dong X , Wang T , Lei Y , Higgins K , Liu T , Curran WJ , Mao H , Nye JA , Yang X . Synthetic CT generation from non-attenuation corrected PET images for whole-body PET imaging. Phys Med Biol. 2019; 64:215016. 10.1088/1361-6560/ab4eb7. 31622962 PMC7759014

[15] Dong X , Lei Y , Wang T , Higgins K , Liu T , Curran WJ , Mao H , Nye JA , Yang X . Deep learning-based attenuation correction in the absence of structural information for whole-body positron emission tomography imaging. Phys Med Biol. 2020; 65:055011. 10.1088/1361-6560/ab652c. 31869826 PMC7099429

[16] Mena E , Lindenberg L , Choyke P . The Impact of PSMA PET/CT Imaging in Prostate Cancer Radiation Treatment. Semin Nucl Med. 2022; 52:255–62. 10.1053/j.semnuclmed.2021.12.008. 35016755 PMC8960055

[17] Fanti S , Goffin K , Hadaschik BA , Herrmann K , Maurer T , MacLennan S , Oprea-Lager DE , Oyen WJ , Rouvière O , Mottet N , Bjartell A . Consensus statements on PSMA PET/CT response assessment criteria in prostate cancer. Eur J Nucl Med Mol Imaging. 2021; 48:469–76. 10.1007/s00259-020-04934-4. 32617640 PMC7835167

[18] Voter AF , Werner RA , Pienta KJ , Gorin MA , Pomper MG , Solnes LB , Rowe SP . Piflufolastat F-18 (^18^F-DCFPyL) for PSMA PET imaging in prostate cancer. Expert Rev Anticancer Ther. 2022; 22:681–94. 10.1080/14737140.2022.2081155. 35603510

[19] Hennrich U , Eder M . [^68^Ga]Ga-PSMA-11: The First FDA-Approved ^68^Ga-Radiopharmaceutical for PET Imaging of Prostate Cancer. Pharmaceuticals (Basel). 2021; 14:713. 10.3390/ph14080713. 34451810 PMC8401928

[20] Ulaner GA , Thomsen B , Bassett J , Torrey R , Cox C , Lin K , Patel T , Techasith T , Mauguen A , Rowe SP , Lindenberg L , Mena E , Choyke P , Yoshida J . ^18^F-DCFPyL PET/CT for Initially Diagnosed and Biochemically Recurrent Prostate Cancer: Prospective Trial with Pathologic Confirmation. Radiology. 2022; 305:419–28. 10.1148/radiol.220218. 35852431 PMC9619197

[21] Isola P , Zhu JY , Zhou TH , Efros AA . Image-to-Image Translation with Conditional Adversarial Networks. 30th Ieee Conference on Computer Vision and Pattern Recognition (Cvpr 2017). Honolulu, HI, USA. 2017; 5967–76. 10.1109/Cvpr.2017.632.

[22] Tao L , Fisher J , Anaya E , Li X , Levin CS . Pseudo CT Image Synthesis and Bone Segmentation From MR Images Using Adversarial Networks With Residual Blocks for MR-Based Attenuation Correction of Brain PET Data. IEEE Trans Radiat Plasma Med Sci. 2021; 5:193–201. 10.1109/Trpms.2020.2989073.

[23] Wahl RL , Jacene H , Kasamon Y , Lodge MA . From RECIST to PERCIST: Evolving Considerations for PET response criteria in solid tumors. J Nucl Med. 2009 (Suppl 1); 50:122S–50S. 10.2967/jnumed.108.057307. 19403881 PMC2755245

[24] Malaspina S , Ettala O , Tolvanen T , Rajander J , Eskola O , Boström PJ , Kemppainen J . Flare on [^18^F]PSMA-1007 PET/CT after short-term androgen deprivation therapy and its correlation to FDG uptake: possible marker of tumor aggressiveness in treatment-naive metastatic prostate cancer patients. Eur J Nucl Med Mol Imaging. 2023; 50:613–21. 10.1007/s00259-022-05970-y. 36161511 PMC9816233

[25] Lodge MA . Repeatability of SUV in Oncologic ^18^F-FDG PET. J Nucl Med. 2017; 58:523–32. 10.2967/jnumed.116.186353. 28232605 PMC5373499

[26] Werner RA , Habacha B , Lütje S , Bundschuh L , Higuchi T , Hartrampf P , Serfling SE , Derlin T , Lapa C , Buck AK , Essler M , Pienta KJ , Eisenberger MA , et al. High SUVs Have More Robust Repeatability in Patients with Metastatic Prostate Cancer: Results from a Prospective Test-Retest Cohort Imaged with ^18^F-DCFPyL. Mol Imaging. 2022; 2022:7056983. 10.1155/2022/7056983. 35283693 PMC8896803

[27] Mena E , Lindenberg ML , Turkbey IB , Shih JH , Harmon SA , Lim I , Lin F , Adler S , Eclarinal P , McKinney YL , Citrin D , Dahut W , Wood BJ , et al. ^18^F-DCFPyL PET/CT Imaging in Patients with Biochemically Recurrent Prostate Cancer After Primary Local Therapy. J Nucl Med. 2020; 61:881–89. 10.2967/jnumed.119.234799. 31676732 PMC9374042

[28] Fourquet A , Rosenberg A , Mena E , Shih JJ , Turkbey B , Blain M , Bergvall E , Lin F , Adler S , Lim I , Madan RA , Karzai F , Gulley JL , et al. A Comparison of ^18^F-DCFPyL, ^18^F-NaF, and ^18^F-FDG PET/CT in a Prospective Cohort of Men with Metastatic Prostate Cancer. J Nucl Med. 2022; 63:735–41. 10.2967/jnumed.121.262371. 34475237 PMC12079750

[29] Gaur S , Mena E , Harmon SA , Lindenberg ML , Adler S , Ton AT , Shih JH , Mehralivand S , Merino MJ , Wood BJ , Pinto PA , Mease RC , Pomper MG , et al. Prospective Evaluation of ^18^F-DCFPyL PET/CT in Detection of High-Risk Localized Prostate Cancer: Comparison With mpMRI. AJR Am J Roentgenol. 2020; 215:652–59. 10.2214/AJR.19.22042. 32755168 PMC8974973

[30] Lindenberg L , Mena E , Turkbey B , Shih JH , Reese SE , Harmon SA , Lim I , Lin F , Ton A , McKinney YL , Eclarinal P , Citrin DE , Dahut W , et al. Evaluating Biochemically Recurrent Prostate Cancer: Histologic Validation of ^18^F-DCFPyL PET/CT with Comparison to Multiparametric MRI. Radiology. 2020; 296:564–72. 10.1148/radiol.2020192018. 32633674 PMC7457947

[31] Rowe SP , Pienta KJ , Pomper MG , Gorin MA . PSMA-RADS Version 1.0: A Step Towards Standardizing the Interpretation and Reporting of PSMA-targeted PET Imaging Studies. Eur Urol. 2018; 73:485–87. 10.1016/j.eururo.2017.10.027. 29132714 PMC6859641

[32] Kinahan PE , Fletcher JW . Positron emission tomography-computed tomography standardized uptake values in clinical practice and assessing response to therapy. Semin Ultrasound CT MR. 2010; 31:496–505. 10.1053/j.sult.2010.10.001. 21147377 PMC3026294

[33] Nyúl LG , Udupa JK . On standardizing the MR image intensity scale. Magn Reson Med. 1999; 42:1072–81. 10.1002/(sici)1522-2594(199912)42:6<1072::aid-mrm11>3.0.co;2-m. 10571928

[34] Ronneberger O , Fischer P , Brox T . U-Net: Convolutional Networks for Biomedical Image Segmentation. Cham: Springer International Publishing. 2015; 234–41. 10.1007/978-3-319-24574-4_28.

[35] Isola P , Zhu JY , Zhou T , Efros AA . Image-to-Image Translation with Conditional Adversarial Networks. Berkeley AI Research (BAIR) Laboratory: UC Berkele. 2017. 10.48550/arXiv.2211.02701.

[36] Cardoso MJ , Li W , Brown R , Ma N , Kerfoot E , Wang Y , Murrey B , Myronenko A , Zhao C , Yang D , Nath V , He Y , Xu Z , et al. Monai: An open-source framework for deep learning in healthcare. 2022. 10.48550/arXiv.2211.02701.

[37] Rosner B , Glynn RJ , Lee ML . Extension of the rank sum test for clustered data: two-group comparisons with group membership defined at the subunit level. Biometrics. 2006; 62:1251–59. 10.1111/j.1541-0420.2006.00582.x. 17156300

