# Supplementary material for: Deep learning-based whole-body PSMA PET/CT attenuation correction utilizing Pix-2-Pix GAN
Source: Oncotarget. 2024 May 7;15:288–300. doi: 10.18632/oncotarget.28583 (PMC11075367; doi:10.18632/oncotarget.28583)
Supplement: Supplementary file 1 [file oncotarget-15-28583-s001.pdf]

Deep learning-based whole-body PSMA PET/CT attenuation correction utilizing Pix-2-Pix GAN

SUPPLEMENTARY MATERIALS

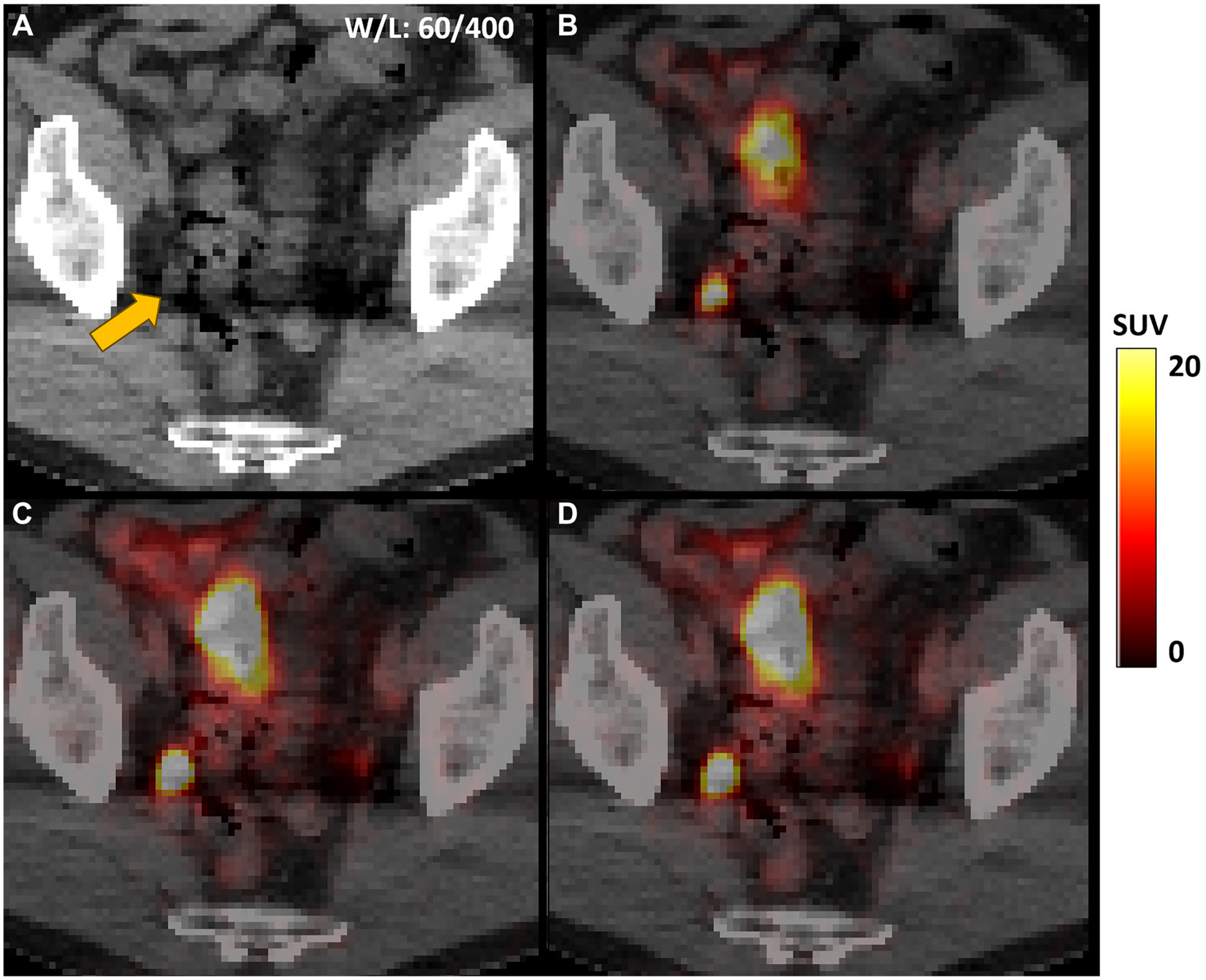

Supplementary Figure 1: Example of AI overestimation of SUV (patient sampled from 1st quartile of mean bias differences). AI-generated PET results shown overlaid on CT. (**A**) Original CT images, pelvic lymph node lesion shown by yellow arrow, (**B**) original AC-PET overlaid on CT (SUV_{max} = 32.62, SUV_{mean} = 15.90). Both AI methods demonstrated overestimation of lesion uptake by SUV_{max} and SUV_{mean} metrics: (**C**) V1-PET on CT (SUV_{max} = 61.49, SUV_{mean} = 31.44); (**D**) V2-PET on CT (SUV_{max} = 61.55, SUV_{mean} = 31.88). Note: CT images were resampled to the voxel resolution of the PET images and shown in unenhanced formatting (no smoothing) for voxel-based visual comparison between methods.

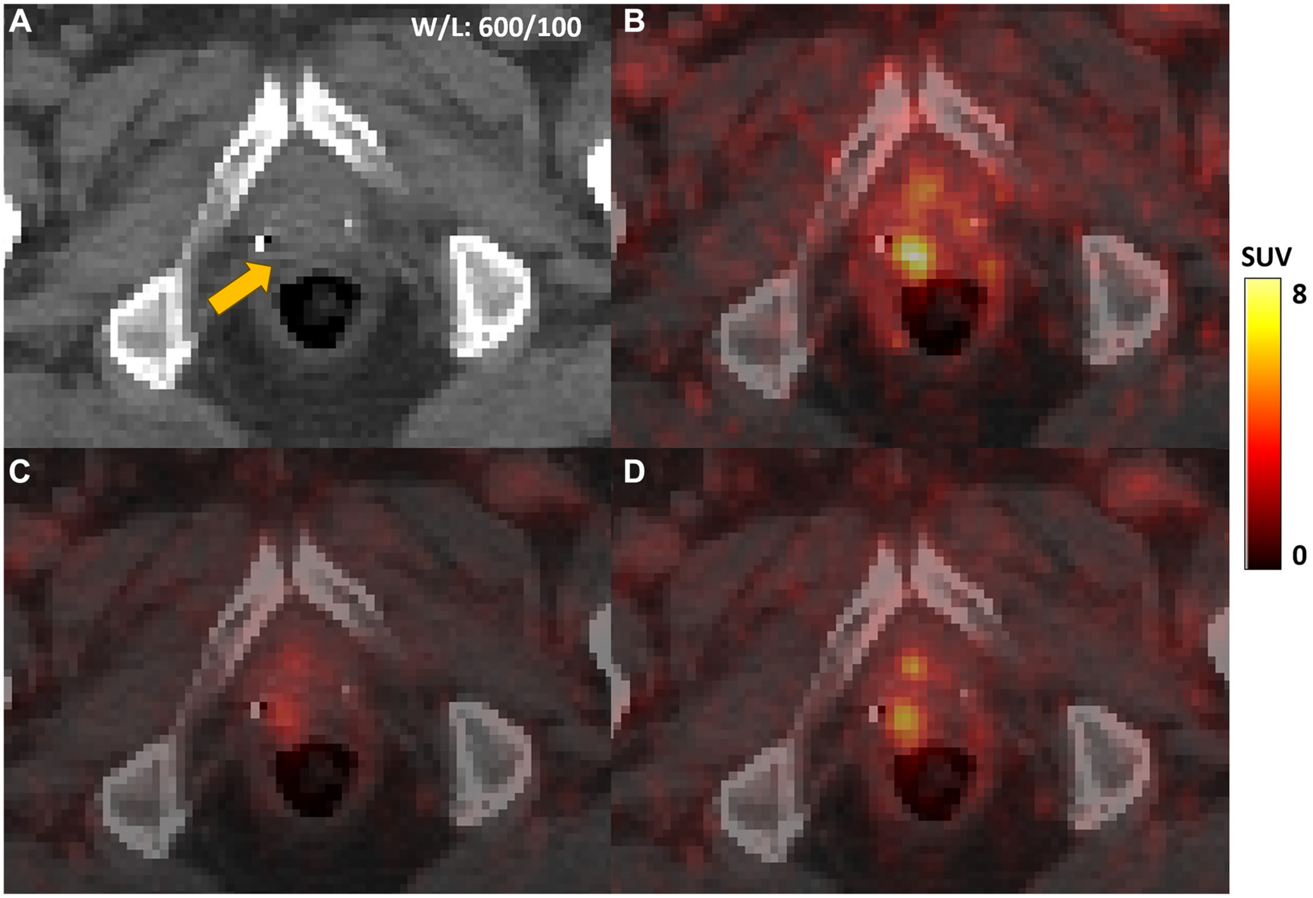

Supplementary Figure 2: Example of AI underestimation of SUV from (patient sampled from 4th quartile of mean bias differences). AI-generated PET results shown overlaid on CT. (**A**) original CT images, intra-prostatic right base peripheral zone lesion shown by yellow arrow, (**B**) original AC-PET overlaid on CT (SUV_{max} =12.45, SUV_{mean} = 6.57). Both AI methods demonstrated underestimation of lesion uptake by SUV_{max} and SUV_{mean} metrics, more notably by fixed threshold (V1): (**C**) V1-PET on CT (SUV_{max} = 3.92, SUV_{mean} = 2.39); (**D**) V2-PET on CT (SUV_{max} = 7.21, SUV_{mean} = 4.60). Note: CT images were resampled to the voxel resolution of the PET images and shown in unenhanced formatting (no smoothing) for voxel-based visual comparison between methods.
